# Active Sensing System with In Situ Adjustable Sensor Morphology

**DOI:** 10.1371/journal.pone.0084090

**Published:** 2013-12-26

**Authors:** Surya G. Nurzaman, Utku Culha, Luzius Brodbeck, Liyu Wang, Fumiya Iida

**Affiliations:** Bio-Inspired Robotics Laboratory, Institute of Robotics and Intelligent Systems, Department of Mechanical and Process Engineering, ETH Zürich, Zürich, Switzerland; University of Vermont, United States of America

## Abstract

**Background:**

Despite the widespread use of sensors in engineering systems like robots and automation systems, the common paradigm is to have fixed sensor morphology tailored to fulfill a specific application. On the other hand, robotic systems are expected to operate in ever more uncertain environments. In order to cope with the challenge, it is worthy of note that biological systems show the importance of suitable sensor morphology and active sensing capability to handle different kinds of sensing tasks with particular requirements.

**Methodology:**

This paper presents a robotics active sensing system which is able to adjust its sensor morphology in situ in order to sense different physical quantities with desirable sensing characteristics. The approach taken is to use thermoplastic adhesive material, i.e. Hot Melt Adhesive (HMA). It will be shown that the thermoplastic and thermoadhesive nature of HMA enables the system to repeatedly fabricate, attach and detach mechanical structures with a variety of shape and size to the robot end effector for sensing purposes. Via active sensing capability, the robotic system utilizes the structure to physically probe an unknown target object with suitable motion and transduce the arising physical stimuli into information usable by a camera as its only built-in sensor.

**Conclusions/Significance:**

The efficacy of the proposed system is verified based on two results. Firstly, it is confirmed that suitable sensor morphology and active sensing capability enables the system to sense different physical quantities, i.e. softness and temperature, with desirable sensing characteristics. Secondly, given tasks of discriminating two visually indistinguishable objects with respect to softness and temperature, it is confirmed that the proposed robotic system is able to autonomously accomplish them. The way the results motivate new research directions which focus on in situ adjustment of sensor morphology will also be discussed.

## Introduction

With the rise of the concept of functional morphology [1], there have been numerous studies in biology which investigates how morphology, i.e. form and structure of organisms and their body parts, contribute to performances and functions in different kinds of environments [2]. Taking sensing as an example, various studies have shown the importance of sensor morphology in transducing stimuli, e.g. mechanical, chemical, or visual, into signals that can be further processed by internal control structure like central nervous system with suitable characteristics [3-5]. In the simplest case, cells sense mechanical stimulus and transduce them into a biochemical signal, in which their cytoskeletal was argued to have an important role [3]. For more complex creatures, it has been found that the spacing of the facets in the compound eyes of house flies is denser toward the front of the animal, compensating for the phenomenon of motion parallax [4]. It has also been shown that variations in both hair densities and hair lengths on wild crickets’ cerci determine their wind sensing sensitivity [5]. 

In order to accomplish different sensing tasks, it is also often necessary to have different interactions between the sensor and the stimuli from the environment through the motion of the sensor. In this case, the sensing system is considered to be active. More fundamentally, active sensing system refers to purposive and information seeking sensory systems [6]. For example, in order to sense different properties of an object, we press our finger to determine softness, stroke a surface to detect texture, or simply statically place our fingers to discriminate temperature [7]. Therefore, while sensor morphology determines the sensing characteristics when the stimuli from the environment are transduced into usable signal, active sensing allows a selection of suitable amount and type of the stimuli.

The interdependence between active sensing via suitable motion and sensor morphology in accomplishing different kinds of sensing tasks has also been discussed in literatures, particularly in active touch sensing system [6,8,9]. For instance, rats adjust their whisking control strategy to accomplish a texture discrimination task due to the changes in the morphology of its vibrissal array [8]. Another experiment also suggests that a specific morphology of the harbor seals whisker causes a near to optimal signal to noise ratio, determining the seals strategy in using their whisker for searching and tracking hydrodynamic trails [9]. A noticeable relationship between active sensing and sensor morphology is shown by web builder spiders. It is well known that certain animals, e.g. spiders, birds, beavers, show structure building behaviours [10]. In the case of spiders, the built web is used for foraging purpose, and therefore involves sensing tasks [11,12]. In order to probe the existence and properties of an unknown target object, i.e. possibly captured prey, spiders perform a radius-pulling behaviour on their web which will change the amount of vibration stimuli over time received by its sensory receptor [11]. Moreover, spiders are also found to alter the morphology of the web in response to exposure to different prey types and traits [12]. In other words, in order to fulfill different task requirements, spiders are able to repeatedly construct passive mechanical structures in situ with adjustable morphology and use it as if it is its part of its own body for active sensing purpose. 

In robotics research, there are several attempts to exploit this interdependence in order to accomplish different kinds of sensing tasks with suitable performance. For example, it has been shown that concurrently evolving the sensors placement and the motion control significantly improve the effectiveness of hexapod robot navigation [13]. In multi-robot setting, the interdependence between sensor morphology and the motion of each robot has also been studied in a context of formation control [14]. Despite the highly motivating efforts, robotic systems are expected to operate in ever more unknown and uncertain environments [2]. While, for instance, there are also researches which focus on the software architecture for realizing adaptive and modular sensing system [15], a significant challenge in this line of robotics research seems to be the technological solution to autonomously and adaptively vary the physical structures to test variations of sensor morphology in situ to handle possible unanticipated task requirements. To the best of our knowledge, all of these sensor morphology researches have been unable to adjust the shape, size and connectivity of the mechanical structures in situ once being fabricated, or they were manually altered with human assistance.

A representative example of robotics research that focuses on the ability and technological solution to autonomously change the robot morphology in situ is known as modular self-reconfigurable robotic system (MSR). The commonly used approach in MSR is to execute a reconfiguration algorithm to rearrange the connectivity of a given number of pre-defined modules equipped with motors and sensors that can connect to or disconnect from the other same type of modules [16]. By varying the connectivity between modules, the robot can transform itself into a different shape most suitable to accomplish the task in hand [17,18]. For example, it has been demonstrated that a robot based on MSR concept is able to generate multiple locomotion gait patterns such as wheel-like rolling or snake-like crawling [18]. However, there are several challenges in the conventional MSR approach [19,20]. Firstly, the pre-defined modules generally require costly mechatronic design. Secondly, the complex connection mechanisms between the modules give a considerable limitation to the flexibility of possible shapes that the system can achieve. Moreover there are still a number of challenges related to the physical constraints of this approach such as weight, motor power density, and robustness in various environments. In order to overcome these problems, alternative approaches are proposed which are capable of fabricating passive mechanical structures, i.e. does not have its own actuation power, by using unconventional materials. One example is a robot which is capable of discharging foam in situ to quickly build doorstop or plate in hazard disposal scenario [19]. Another example focuses on the use of Hot Melt Adhesives (HMA) to accomplish passive gripping based on additive fabrication concept [20]. Outside robotics research, the use of additive fabrication has also been proposed in rapid prototyping research area [21,22], and even as a full scale manufacturing solution [23,24]. 

**Figure 1:**
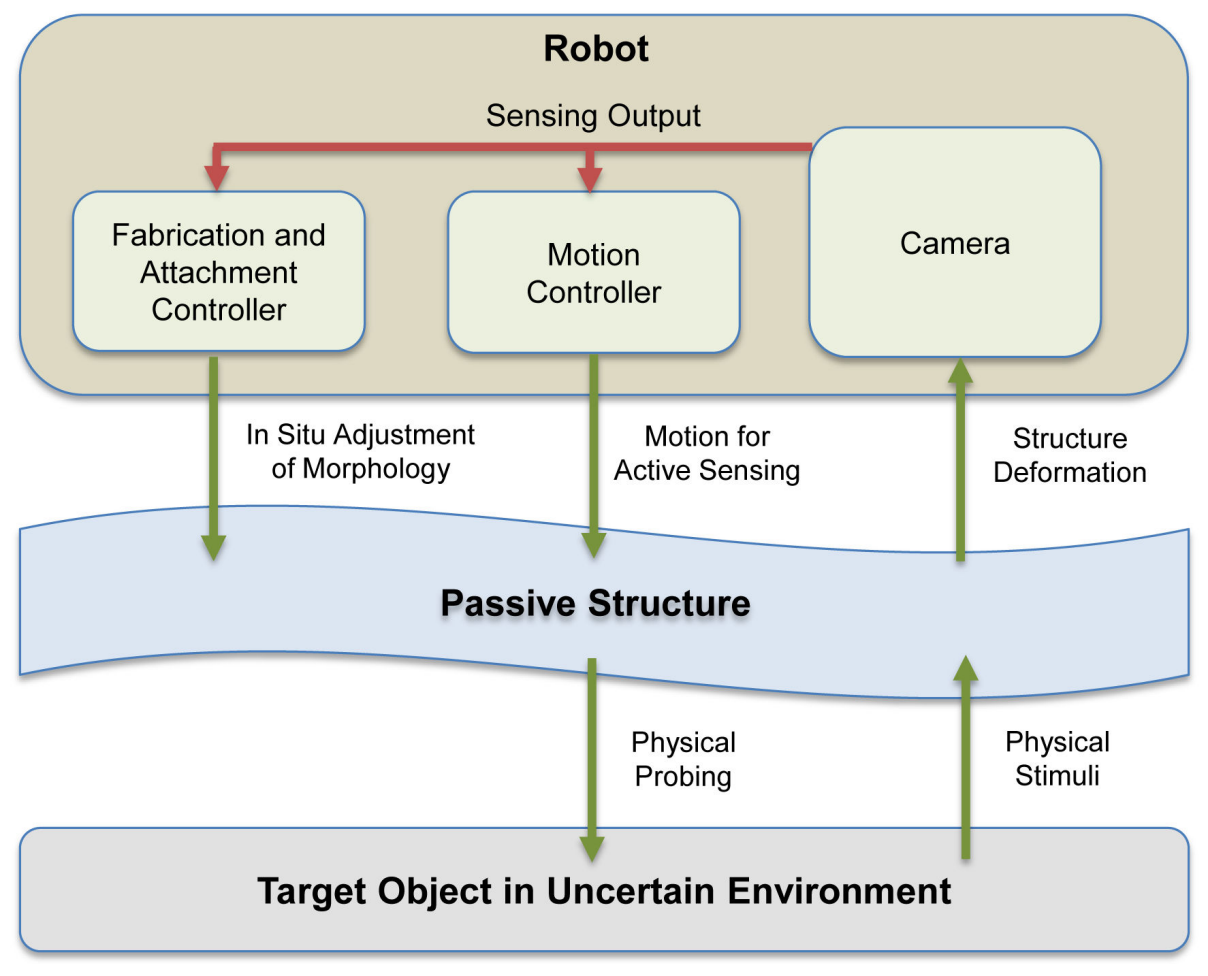
Basic concept of robotics active sensing system with in situ adjustable sensor morphology. In order to sense a possibly unknown target object in uncertain environment, a passive mechanical structure is used by a robotic system to probe the object via suitable motion. A camera will observe this physical interaction and transduce the deformation of the structure due to the arising physical stimuli into useful geometrical information as a sensing output. Based on the output, a fabrication/attachment controller and a motion controller can decide the necessity and the way to adjust the sensor morphology in situ, i.e. the shape, size and connection of the mechanical structures, and/or the suitable motion to initiate different physical interactions. The red lines correspond to the sensing output obtained from the camera, while the green lines correspond to the involved processes during the physical interactions between the robot and the target object.

The main goal of this paper is to propose a concept and technological solution of robotics active sensing system which is able to adjust the sensor morphology in situ, and confirm its advantage to accomplish different sensing tasks with particular requirement, i.e. to sense different physical quantities with suitable sensing characteristics. The conceptual figure of the proposed system is shown in Figure 1. A robotic system is equipped with the ability to repeatedly fabricate and attach/detach passive mechanical structures with suitable morphology to its own body. In order to use the structure properly to probe a target object in unknown or uncertain environment, the robot should also be able to perform active sensing via suitable motion. These two abilities are controlled by the fabrication and attachment controller and the motion controller respectively. A camera is chosen as the robot’s only built in sensor. The camera will observe the deformation of the structure due to the physical interaction with the target object and provide the sensing output, i.e. the geometrical information describing the deformation, for the controllers. A camera is also chosen because it can transduce the physical interaction into the sensing output independent of the possible attachments/detachments between the physical structure and the robot end effector. If a task requirement is not yet fulfilled, i.e. sensing particular physical quantities with desirable sensing characteristics, the controllers can adjust the sensor morphology, i.e. the shape, size and connection of the mechanical structures, and/or the motion to initiate different physical interaction. 

In order to realize the concept, the proposed technological solution is to use a robotic arm that is able to repeatedly fabricate, dispose and manipulate passive mechanical structures for sensing purpose. Hot Melt Adhesive (HMA) is chosen as the material for the mechanical structure. The attractive properties of HMA lie in the fact that it is thermoplastic and thermoadhesive. The material can be transformed between solid and liquid phases by increasing/decreasing material temperature, and the material in liquid phase exhibits adhesive property, while it forms bonding when solidified by cooling. More specifically, it is hypothesized that: (1) the thermoplastic and thermoadhesive nature of HMA will enable the system to repeatedly fabricate different mechanical structures and integrate them in situ to adjust the sensor morphology and therefore the sensing characteristics (2) once the sensor morphology is adjusted, active sensing via suitable motion can be executed in order to obtain suitable amount and type of desired stimuli (3) additionally, due to the use of a robotic system, these two processes can be executed autonomously. 

The rest of the paper is organized as follows. The Materials and Method Section will explain the used robotic platform, as well as the hardware and software implementation. The hypotheses will be confirmed in the Results section. More specifically, firstly, it will be verified whether suitable sensor morphology and active sensing capability enables the system to sense different physical quantities through the stimuli, with desirable sensing characteristics. In this paper, softness and temperature sensing tasks are chosen as case studies. The second part of the Results section will demonstrate the capability of the system to autonomously accomplish case studies of discriminating two visually indistinguishable objects with respect to softness and temperature, given the suitable design parameters. The discussion and conclusion of the research are made in the Discussion section.

## Materials and Methods

In this section, we will describe the proposed robotic active sensing system which consists of the hardware platform and control architecture. We will also explain how the HMA mechanical characteristics lead to the ability to adjust the sensor morphology in situ and the benefit of this ability in coping with uncertain environments. 

### Hardware Platform

**Figure 2:**
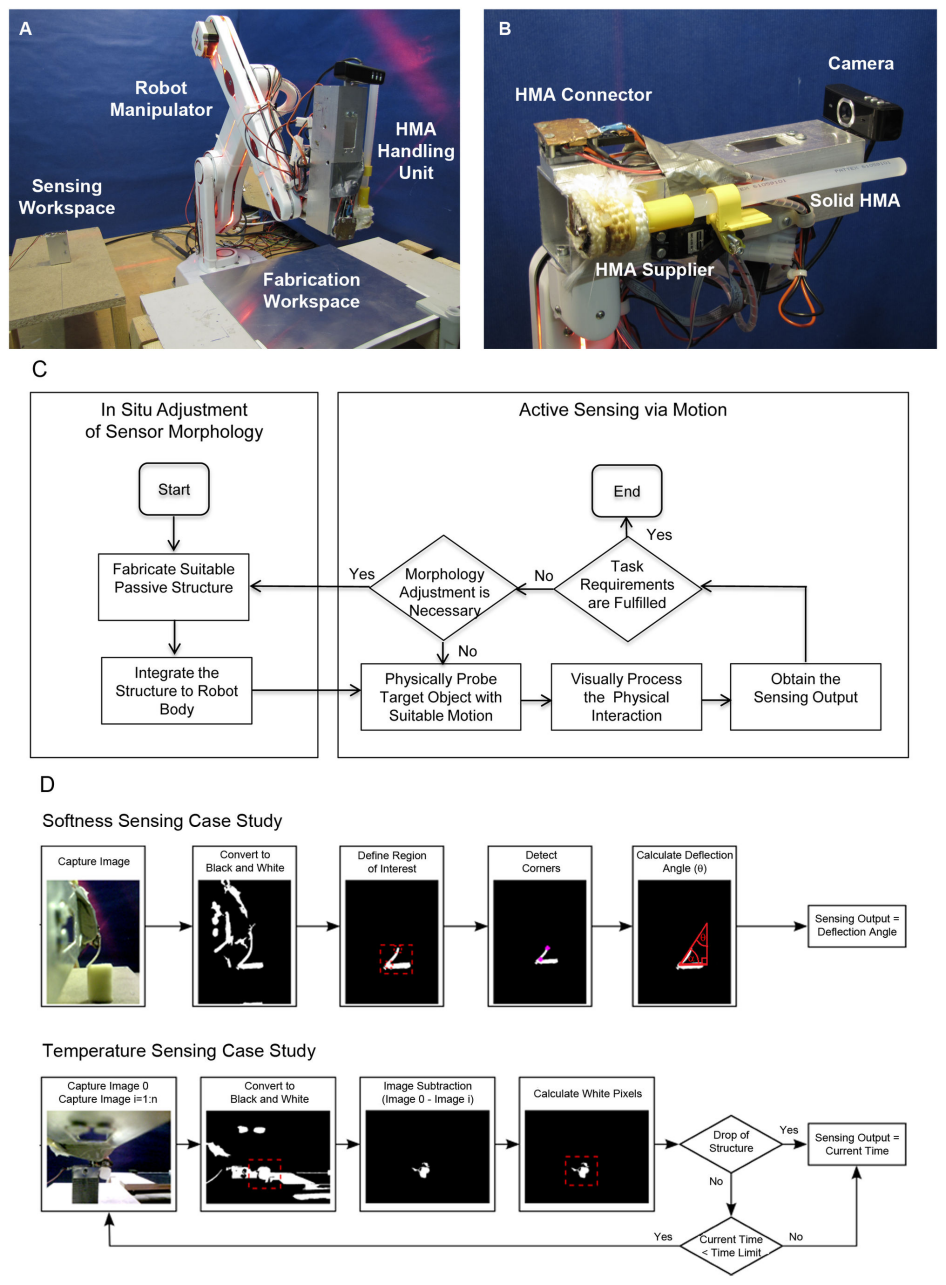
Hardware and software implementation of the proposed concept. (a) Complete workspace of the experiment which includes a robot manipulator equipped with HMA handling units on its end effector (b) The robot’s end effector which is composed of a solid HMA block which is fed to HMA supplier. Fabricated HMA units can be connected to HMA connector. A camera is mounted to perform visual processing tasks during sensing. (c) Software implementation of the proposed approach which is composed of two main parts: the in-situ adjustment of the sensor morphology, and the active sensing via motion (d) Flowchart showing the visual processing algorithm used for softness and temperature case studies.

The proposed hardware platform for realizing the concept in Figure 1 is shown in Figure 2(a) and 2(b), consisting of a robot manipulator and other relevant components, which can be explained as follows. 

The main body of the platform is a commercially available 5-axis robot manipulator (R12 firefly, ST robotics, UK) which is fixated on the ground as shown in Figure 2(a). The setting enables the end-effector, equipped with HMA handling unit, to be precisely positioned within the spherical reachable range of the end-effector with a radius of 500 mm. Two workspaces are prepared within the reach of the end-effector, i.e. (1) a fabrication workspace where the robot will fabricate the HMA based structure and attach it to its own body (2). a sensing workspace where the robot will perform the sensing task. 

The second component of our platform is the HMA handling unit shown in Figure 2(b), which is fixated at the fifth joint of the manipulator. The HMA handling unit is made of aluminum-based housing, and equipped with commercially available web camera with resolution 640x480 [pixels], as well as HMA Connector and HMA Supplier. 

The HMA Connector has a function of heating and cooling the connecting surface which is used to connect to and disconnect from the HMA based structure, and has three-layer structures. The outmost layer is a copper-based plate (25x30 [mm^2^] rectangular surface), selected due to its high heat conductivity and bonding characteristic. The middle layer is a commercially available Peltier element (TEC1-01703, Centenary Materials, China) closely attached to the copper plate. The device acts as a heat pump such that one side of the device is cooled down while the other is heated up, when an electric current is applied. The third layer consists of a heat sink, which is attached on the other side of the Peltier element. 

The HMA Supplier is designed to transform solid-phase HMA sticks into liquid-phase HMA flow. One end of this device has a CNC-manufactured aluminum part which functions as a HMA melting cavity and supply nozzle. The melting cavity is surrounded by heating resistors (6 x 10 [Ω] in parallel configuration) such that the cavity can be heated to a temperature of around 150 [°C] that turns HMA into liquid phase. The cavity temperature is regulated by a simple on-off controller, and the feedback is provided by a thermo sensor (CON-TS-NTC-202, Hygrosens, Germany) mounted near the nozzle. The melting cavity is also covered by a HMA Support Tube made of heat-resistive silicon, designed to hold a solid-phase HMA without too much friction such that solid HMA can be smoothly pressured into the cavity. Alongside of the solid-phase HMA, a servomotor (Modelcraft MC-630MG) is installed to exert a controlled force on the HMA.

### Control Architecture

The proposed concept can be practically implemented as a series of motor commands from a host computer to the robot platform. A MATLAB script was executed in the host computer to communicate with various microcontrollers and sensors, i.e. microcontrollers to regulate motor actions of HMA Connector and Suppliers, microcontrollers to control the motion of the robot platform, and a vision sensor. Therefore, the two controllers shown in Figure 1, i.e. the fabrication /attachment controller and the motion controller, can be executed in a fully centralized manner. 

Figure 2(c) shows the software implementation in a flowchart form. The flowchart can be conceptually divided into two parts, i.e., “In Situ Adjustment of Sensor Morphology” and “Active Sensing via Motion”. In the first part, the robot manipulator explained in the section “Hardware Platform” will fabricate the passive mechanical structure with suitable shape and size through additive fabrication of HMA and attach it properly to the robot end effector. After the sensor morphology is adjusted, the second part controls the robot manipulator to approach an object to be sensed in order to probe the object and initiate a physical interaction between the structure and the object via suitable motion. A visual based calculation is also executed during the process to measure different physical quantities as the sensing output. Finally, based on the generated sensing output, the controllers will decide whether it is necessary to adjust the motion and/or the sensor morphology, or whether the task requirements are already met such that the sensing process can be ended. In this paper, the focus is on the realization of the whole system and therefore the control of the robot behaviour is simply predefined for each case study.

Figure 2(d) shows the visual processing in a more detailed manner. The visual processing was designed based on the standard vision toolbox in the MATLAB program environment. More specifically, depends on the sensed physical stimuli, the system starts with capturing a raw gray-scale image, converting it to a binary image, cropping the region of interest, and estimating the deformation of the sensor due to the interactions with the target object. Therefore, the camera transduces the physical quantities into geometrical information which describes the deformation of the structure due to the arising stimuli. For each case study, this process runs automatically and uses the exact same algorithm to obtain the necessary information.

### HMA Mechanical Characteristics for In Situ Adjustment of Sensor Morphology

HMA is a mixture of polymer and other ingredients such as wax and resin. The material has a highly interesting property, with which it is able to repeatedly transform between adhesive fluid and solid phases by controlling the temperature. Typically HMA exhibits three phases depending on its temperature: (1) At room temperature (*Tr* = 25 [°C]), HMA is in solid phase and has no adhesiveness (2) At higher temperature around its softening point, *Ts* (typically equals to 60°C) HMA becomes viscoplastic and adhesive (3) At an even higher temperature between *Ts* and melting point (Tm = 150 [°C]), the material transforms into a low-viscosity fluid. The value of *Ts* and *Tm* varies depending on the ingredients of HMAs. It is particularly important to mention that HMA at room temperature has a tensile strength (typically around 1-10 [MPa]) sufficient to form a large variety of reasonable mechanical structures that can be used for sensible robotic tasks. 

In the later stage of this paper, it will be shown how the mechanical characteristics of HMA will be used in the fabrication processes of the mechanical structures. For fabrication process, we make use of the so-called additive fabrication method, in which a thin string of soft/liquid HMA is placed on a fabrication table such that the deposited strings form a free form solid structure when they are solidified. The important mechanical characteristic of HMA, therefore, is its viscosity that can be precisely controlled such that the fabrication process constructs fine structures. In our approach, we employed a heating device that has a small nozzle attached, where a heated and pressurized HMA can be extruded as a string. Because of its adequate viscosity that can be controlled through material temperature, we are able to reliably control the HMA strings diameter as precise as 1 [mm]. A more thorough explanation about the relationship between the diameter and nozzle velocity, how the velocity is controlled, and other technical details, can be found in [20]. Here, it is adequate to note that the used nozzle velocity in this paper is 2 [mm/s], causing the diameter of the string to be 1.5 [mm]. 

Based on the explained properties, it is hypothesized that the thermoplastic and thermoadhesive properties of HMA can enable in situ adjustment of sensor morphology, i.e. HMA enables fabrication of a variety of mechanical structures with different size and shape, which can easily be attached / detached to the rest of the robot’s body for sensing purpose. The relationship between the temperature and the size of attachment area between the mechanical structure and robot’s end effector will be explained further in the next section. 

### HMA Mechanical Characteristics for Sensing

Based on the assumption that it is possible to fabricate and easily integrate the mechanical structure with the robot arm in situ, the next step is to verify whether a suitable physical interaction, i.e. physical probing and the arising stimuli, can be initiated for sensing task based on HMA mechanical characteristics. Here, the chosen task for the system is to discriminate visually indistinguishable objects with respect to different physical quantities, i.e. softness and temperature. They are chosen because therefore the role of two important mechanical characteristics of HMA, i.e. tensile strength and thermoadhesion, can be effectively tested. The designed physical interaction and the way different sensor morphologies may affect the sensing characteristics will be described as follows. 

**Figure 3:**
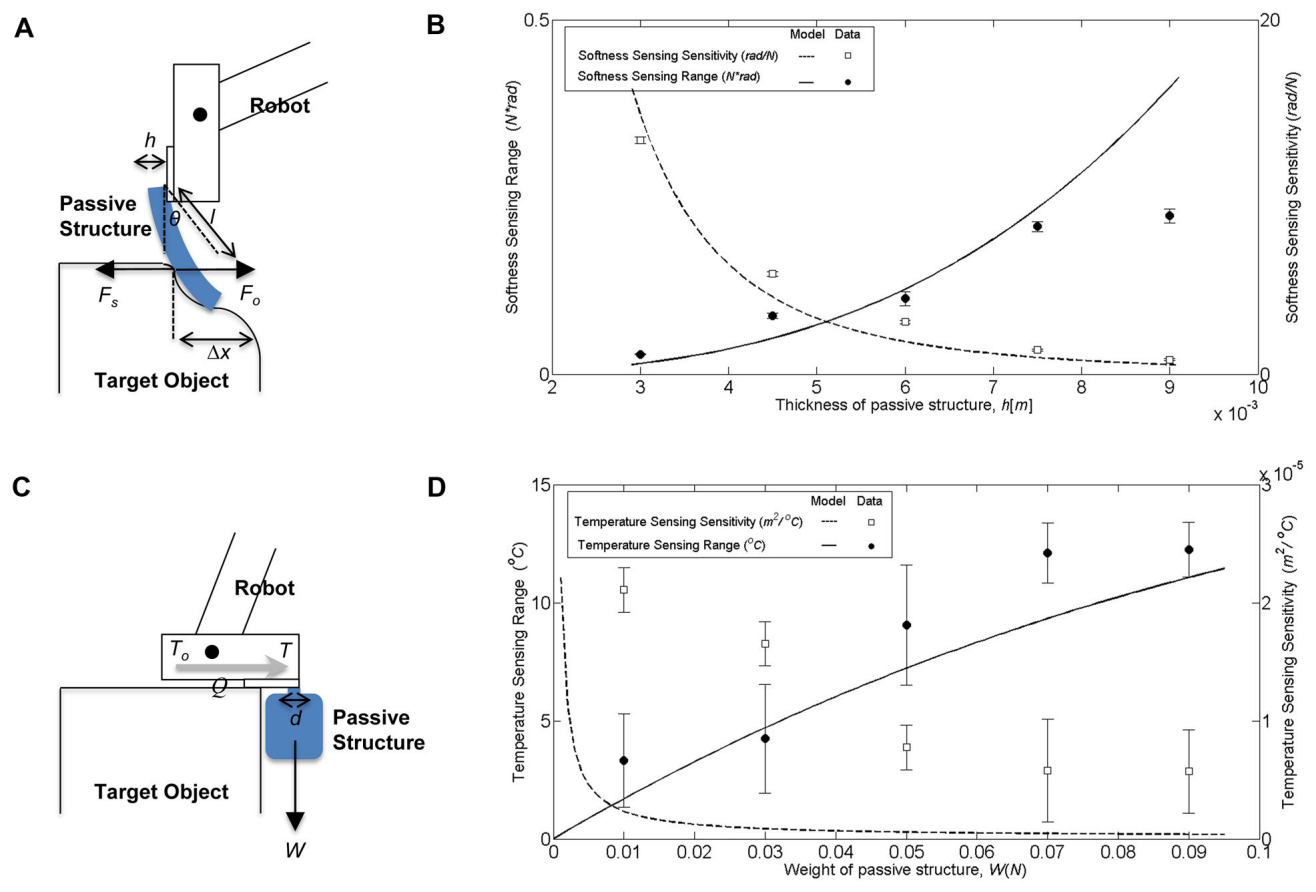
Different physical interactions and sensing characteristics enabled by adjusting the sensor morphology, and purposive motion, in situ. (a) model of the physical interaction for discriminating the softness of the target object (b) corresponding sensing characteristics, i.e. range and sensitivity (c) model of the physical interaction for discriminating the temperature of the object (d) the corresponding sensing characteristics (note: the standard deviation for temperature sensing range is divided by two for the sake of clarity).

In order to discriminate the softness of an object, it is necessary to be able to distinguish the amount of force exerted by the object when pushed by the sensor. The simplest way to achieve it is by having a comparatively elastic cantilever, and to estimate the force through the deflection of the beam, namely, by estimating the value of force *F* [N] through the deflection *θ* [rad] by using function *f* as shown in (1a). Figure 3(a) illustrates the situation, where *F* is the force applied to the fabricated stick as a reaction to the force *F*_*s*_ applied to the object. By estimating the value of *F* = *F*_*s*_ through *θ*, if the object is assumed to be linearly elastic, the softness the object can be estimated by using Hooke’s law if *∆x* is known (The Hooke’s law states that the force required to extend or compress a spring by some distance is linearly proportional to the distance). 

A tension test to see the relationship between the tensile stress σ [Pa] and strain ε [-] was performed with HMA string. It is found that for small strain (ε < 0.2), the stress-strain relationship is linear with a Young modulus *E* of approximately 8.9 [MPa]. Based on the beam theory, within the linearly elastic region, the function that relates *F* and *θ* can be simplified as a linear one which depends on the value of the length of the cantilever l [m], the Young modulus *E* [Pa], and the second moment of area *I* [m^4^] [18]. Therefore, function *f* can be written as shown in (1b). It must however be noticed that there is a value *θ*_*max*_ where the linear relationship between *F* and *θ* still holds.

F=f(θ) (1a)

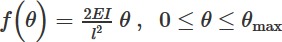
(1b)

Due to the additive fabrication process, the second moment of area can be modeled as a rectangle with width *d* [m] and thickness *h* [m] as shown by (2), which modifies (1) to become (3). 

$$I=\frac{dh^{3}}{12}$$ (2)

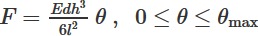
(3)

The effect of the sensor morphology and properties of HMA on the sensing characteristics can be described as follows. Due to the linear relationship assumption between *F* and *θ*, the sensing range of the sensor, i.e. the maximum value of force can be accurately estimated by observing the value of *θ*, is limited. The sensing range of the sensor *R*_*F*_ [Nrad] therefore simply equals to (3) with *θ* = *θ*_*max*_. Furthermore, the sensitivity of the sensor *S*_*F*_ [rad/N], i.e. the derivative of *θ* to *F*, can be easily obtained as shown in (5).

$$R_{F}(d,l,h)=\frac{Edh^{3}}{6l^{2}}\theta {|}_{\theta =\theta_{\max}}$$ (4)

$$S_{F}(d,l,h)=\frac{d\theta }{dF}=\frac{6l^{2}}{Edh^{3}}$$ (5)

It can be seen that increasing the value of *d* and *h* will increase the sensing range while decreasing the sensitivity, while increasing the value of *l* will have the opposite effects. Figure 3(b) shows how these two characteristics change by adjusting the thickness *h*, with d=3 [mm] and *l*=4.5 [cm], attached to the robot end effector at a 3x3 [mm^2^] area. Based on the figure, it can be seen, for example, that in order to measure the softness of a relatively hard object, a larger value of *h* may prove to be beneficial as it leads to a larger sensing range to measure a significant value of force exerted by a hard object. 

Owing to the thermoadhesive property of HMA, its mechanical characteristics can also be exploited for sensing temperature. The physical interaction for the case of temperature discrimination is shown in Figure 3(c). By touching the object with its end effector, due to heat conduction *Q*, temperature *T* will increase as *T*_*o*_ is increased. As a result, the fabricated mechanical structure will be detached from the robot’s end effector. By collecting experimental data, the relationship between temperature *T* and bonding strength *B* can be approximated by exponential function as shown by (6), where the relationship among bonding strength (*B*), weight (*W*) and the attachment area (*A*) is explained by (7). *m* is the mass of the system, *g* is gravitational acceleration, and *d* is the width of the area. The bonding area can be assumed as a square with width *d*, while *k*_*T1*_ and *k*_*T2*_ are the constants included in the equations. From experiment, the value of *k*_*T1*_ and *k*_*T2*_ are 7.75x10^1^ [°C] and 4.70x10^-5^ [N^-1^m^2^] respectively. Based on (6), the resolution as well as the maximum and minimum value of the temperature which can be sensed therefore depends on the weight *W* [kg] of the built HMA based structure and the size of the attachment area *A* [m^2^] between the structure and the robot end-effector. Due to the designed physical interaction, the built mechanical structure will be detached once *T* reaches the value of *T_o_.*

$$T=k_{T1}\exp(-k_{T2}B)$$ (6)

$$B=\frac{W}{A}=\frac{W}{d^{2}}=\frac{mg}{d^{2}}$$ (7)

The most difficult challenge to realize the physical interaction shown by Figure 3(c) is to attach the mechanical structure with an arbitrary value of area *A*. It will be technically very difficult, for example, to attach the unit with a very small A. On the other hand, having a very large value of *A* is not feasible due to the time and energy consumption. In this second case study, therefore the temperature measurement is limited by the minimum and maximum value of *A*. Here, the temperature sensing range is defined by the sensed temperature for the same mechanical structure weight when the attachment area is changed from 3x3 [mm^2^] to 4x4 [mm^2^] as shown by (8). The sensitivity is defined as the derivative of the attachment area *A* over the temperature *T*, shown by (9). The sensing range *R*_*T*_ [°C] and sensitivity *S*_*T*_ [m^2^/°C] figure for sensing object temperature as a function of the weight is shown in Figure 3(d). Here, it can be clearly seen that the range of the sensor and sensitivity can be tuned by adjusting weight of the designed mechanical structure. A larger weight, for example, will have a wider sensing range at the cost of the sensitivity.

$$R_{T}(W)={T|}_{A=A_{\max}}-{T|}_{A=A_{\min}}$$ (8)

$$S_{F}(W)=\frac{dA}{dT}=\frac{A^{2}\exp(k_{T2}(\frac{W}{A}))}{Wk_{T1}k_{T2}}{|}_{A=A_{\min}}$$ (9)

## Results

### Verification of the Model

In this section, we will verify the models proposed in the Materials and Method section, and describe how different sensor morphology enables the system to sense different physical stimuli with tunable sensing characteristics.

For the force sensing case study, experimental data were collected by varying the thickness of the HMA based cantilever, and measured the corresponding deflection angle and reaction force from the object by using force gauge with resolution 0.05 [N]. Each experiment was performed for 10 trials. The *R*_*F*_ data collected from the experiments are defined as the maximum value of force which can still be measured without having the root mean squared error between the real, obtained, value and the linear model exceeding 0.005. The S_*F*_ data collected from the experiments are defined as the gradient of the line that best fits the relationship between the measured angle and force, for each value of *h* , i.e. thickness of the structure. The dots in Figure 3(b) show the experiment data, plotted in the same figure as the model explained in the previous section. 

For the temperature sensing case study, experimental data were collected by varying the mass of a cylindrical shape mechanical structure to 1,3,5,7 and 9 [g] for attachment area of 3x3[mm^2^] to 4x4 [mm^2^]. The value of *R*_*T*_ and *R*_*S*_ based on the collected data are shown by the dots on the right picture of Figure 3(d). The number of trials for each experiment was five. 

Based on the collected data in each case study, it can be seen that the data adequately conform with the proposed model. Therefore, the hypotheses that the proposed system is able to sense different physical quantities with suitable sensing characteristics by adjusting the sensor morphology can be confirmed. 

### Demonstration of the Autonomous Capability of The System

After confirming the advantage of the ability to adjust the sensor morphology in situ, in this section we implement the system to autonomously accomplish case studies of discriminating visually indistinguishable objects with respect to softness and temperature autonomously (please also refer to Video S1).

**Figure 4:**
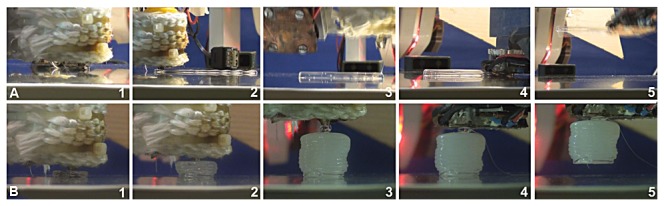
Implementation of autonomous in situ adjustment of sensor morphology. The whole process includes the construction of the unit which is followed by the gripping of that unit by the HMA connector on the end effector of the robot. (a) Construction and attachment process of the mechanical structure used for discriminating the softness of objects. (b) Construction and attachment process of HMA mechanical structure for discriminating the temperature of objects. Please also see Video S1.

Given the design parameter, Figure 4 shows the mechanical structure fabrication for discriminating (a) softness, and (b) temperature. As explained in the Materials and Method section, the chosen shape of the mechanical structure for discriminating the object softness is a cantilever with designed width, length and thickness. As for discriminating the temperature, the chosen shape is a cylindrical shape such that each layer adds the weight of the unit.

The process always starts with additive fabrication, in which the robot manipulator follows a given trajectory while HMA Supplier is controlled to continuously extrude a liquid HMA string from the nozzle. As shown in Figure 4(a1-2), the nozzle moves to the lateral directions back and forth with a certain given length, which resulting in a stick-like structure when the HMA is solidified at the room temperature. In contrast, the cylindrical structure requires a spiral trajectory of the nozzle to make a layer of disc-like structure, which is then accumulated vertically as shown in Figure 4(b1 - 2). 

Once the additive fabrication is completed, for integrating the fabricated mechanical structure with the rest of the sensing system, the HMA Supplier provide a drop of fluidic HMA that is used for bonding between the HMA Connector and the fabricated structure as shown in Figure 4(a3 - 4) and (b3 - 4). After the cooling period of bonding, the robot is now able to separate the fabricated structure from the fabrication workspace to lift up the structures as shown in Figure 4(a5) and 4(b5). 

For the experiments of sensing performance, we constructed six distinctive HMA-based mechanical structures, i.e. three for softness and the other three for temperature. For softness sensing, all three structures have the length of *l*=4.5 [cm] while we selected three different thicknesses, i.e. 3, 4.5 and 6 [mm]. The fabrication processes of these structures can be simply determined by the number of lateral motions repeated. As for the temperature sensing, we tested three different masses of cylindrical structures, i.e. 2.3, 3.6, and 7.7 [g], which can be determined a number of disc layers accumulated vertically. For all fabricated structures, the robot always makes use of bonding area of 3x3 [mm^2^].

Once the fabrication of mechanical structure is completed, the controller starts the Active Sensing Process shown in Figure 1. The sensing process can be roughly decomposed into two sets of actions, i.e. motor control of the robot manipulator and visual image processing. 

The robotic manipulator was programmed to execute a single trajectory for each of the tasks. Assuming that the target object is always located at the same location with respect to the robot’s coordinate system, the manipulator operates an open-loop position control to place the position of the end effector. In case of softness sensing the robot manipulator places its end-effector at the corner of target sensed block and the fabricated stick-like structure is pushed against the block. For temperature sensing, the end-effector motion is programmed to place the HMA Connector to touch the edge of sensed object such that the surface of HMA Connector could transfer the heat of object to the bonding area where the mechanical structure is attached.

As soon as the motion control has been executed, the visual processing starts, and the process involved in the vision algorithm is illustrated in Figure 2(d). As explained in the Control Architecture section, all of the visual processing was designed based on the standard vision toolbox in the MATLAB program environment. More specifically, for each the physical quantity to be sensed, the system starts with capturing a raw gray-scale image, converting it to a binary image, cropping the region of interest, and estimating the deformation of the mechanical structure due to the interactions with the target object. For the softness sensing, the system calculates the result from ten pictures for every trials, while for the temperature sensing, the visual system keeps comparing the current and previously captured picture until a predefined time. As the output of visual processing, the softness sensing gives angle values of the stick-like structure deflected by the reaction force of the sensed object, and the temperature sensing provides the duration until the cylindrical structure disappears from the visual field.

**Figure 5:**
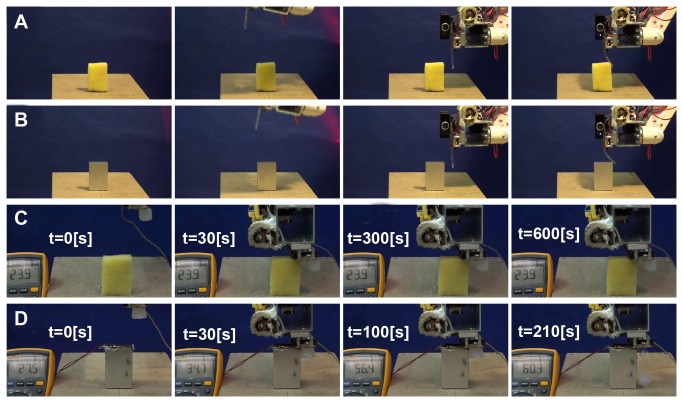
Implementation of autonomous active sensing via suitable motion. Each sequence starts with the approaching of the HMA mechanical structure onto the object, followed by the corresponding physical interaction monitored by a visual processing algorithm (a) Image sequences showing the softness discrimination experiment on a sponge block where the deflection angle of the HMA string is calculated. (b) Image sequence of the same experiment for an aluminum block (c) Temperature sensing of the same sponge block which is at a room temperature. (d) Temperature sensing on the aluminum block which is equipped with heaters to adjust the temperature of the object to a fixed level approximately at 120°C. The temperature sensor attached to the connection surface between the HMA connector and the aluminum block shows the gradual increase from room temperature to 59.3. Please also see Video S1.

The proposed approach is tested as a discrimination task on different object pairs for two modalities; for softness, robot should be able to discriminate a sponge from an aluminum block, for temperature, the sponge is compared with an aluminum block at 120 [°C]. Figure 5 shows the image sequences captured from different sensing tasks. In Figure 5(a) and (b), the different deflection angle is used to discriminate the stiffer aluminum block from the softer sponge block. In Figure 5(c), the robot has to wait for 600 [s], the time threshold, because the fabricated mechanical structure cannot drop at room temperature. In Figure 5(d), the robot detects the drop of the mechanical structure after 210 [s]. 

The experiments were repeated with three different sizes of each mechanical structure for every physical quantity each of them has 10 trials of experiment. Experimental results are listed in Table 1. It can be seen that the discrimination task was always successful for softness detection as the deflection angle in sponge was always smaller than in aluminum. As expected, the aluminum exerts a larger value of reaction force, shown by a large value of *θ*. The larger the thickness, the large the difference becomes, which is caused by the increasing stiffness of the mechanical structure with respect to sponge. It must however be noticed that in order to measure the exact value of the force, the resulting value of *θ* is already outside the sensing range based on the derived model. This can be solved by either using a thicker cantilever, or program the robot to push it for a less distance. 

**Table 1:** The summary of the experiment result for autonomous discrimination task showing the relationship between the sensed quantities, the corresponding fabricated passive mechanical structure and its dimension, the resulting sensing output, and the discrimination rate.

Physical Quantity /Mechanical Structure	Mechanical Structure Parameter	Sensing Output		Discrimination
	(*h* = thickness, *W* = weight)	(*θ* = bending angle, *D* = time to detach)		Rate (%)
		Object 1	Object 2	
Softness / String	*h* = 3 [mm]	*θ* = 33.71±3.62	*θ* = 43.60±1.56	100
	*h* = 4.5 [mm]	*θ* = 26.64±2.78	*θ* = 43.71±2.16	100
	*h* = 6 [mm]	*θ* = 22.28±3.33	*θ* = 42.13±2.28	100
Temperature /	*W* = 2.3 [g]	*D* = 600 [s]	*D* = 600 [s]	0
Cylinder	*W* = 3.6 [g]	*D* = 600 [s]	*D* = 253.7±137.1 [s]	90
	*W* = 7.7 [g]	*D* = 600 [s]	*D* = 373.1±182.7 [s]	70

Object 1 is always sponge under room temperature. Object 2 is aluminium under room temperature and heated up aluminium for softness and temperature discrimination respectively.

Results for discrimination on temperature detection show that for small weights, the robot fails to discriminate two different surface temperatures as the mechanical structures do not drop before the waiting time threshold as predicted by the model. When the transducing unit weight is increased, as expected, the discrimination rate increases. However, this did not occur when the weight is kept increased. This flaw may be caused by the imprecision of the connection area during attachment process. While the assumed surface area is 3x 3 [mm2], only 1 [mm] of fault can affect the results in an exponential way due to (6).

## Discussion

In this paper, we have suggested a concept and technological approach of active sensing in robotics system with in-situ adjustable sensor morphology. The approach taken is to use a robotic system that is able utilize thermoplasticity and thermoadhesiveness of HMA to repeatedly fabricate mechanical structure with different shape and size, and easily attach/detach them to robot’s end effector the rest of the system with different configurations, most suitable to the task. By reviewing the studies of biological systems, we have also argued that the proposed system is advantageous for sensing tasks in uncertain environments with possible unanticipated task requirements.

The chosen task for the system is to sense different physical quantities, i.e. softness and temperature, with desirable sensing characteristics. In order to accomplish the task, a suitable physical interaction model is proposed. It has been shown that by having suitable sensor morphology, along with active sensing capability, the physical quantities can be sensed with desirable sensing characteristics. To further confirm the efficacy of the system, it is also shown that the system is able to autonomously accomplish a task of discriminating two visually indistinguishable objects with respect to softness and temperature. 

As the robot is able to adjust the sensor morphology in situ, the proposed concept may play an important role for robots that are aimed to work in ubiquitous terrains, in order to handle possible unanticipated task requirements in unknown and uncertain environments. Our group is also working on embedding a recycling mechanism on the robot which aims to increase the capacity of glue supply and introduce a new level of autonomy in means of keeping track of the fabricated, damaged or detached mechanical structures and putting them back into use for further tasks. Furthermore, as another future work, it is also interesting to explore additional pre-built sensors that act as the sensor receptor aside from vision, as well as investigating more precise models used during the sensing. 

It is also worth mentioning that the proposed concept initiates alternative research directions as compared to the classical approach of sensing, i.e. having many prebuilt sensors with fixed morphology. The classical approach, for example, generated a lot of interests in the sensor fusion research area. This approach, on the other hand, motivates researches on rapid fabrication related technologies, as well as on how to embed cognitive ability to the system in order to increase the autonomy in deciding when it is necessary to adjust the sensor morphology and/or the motion, as well as designing the suitable sensor morphology depends on the task requirement. There are, for example, quite a number of works whose long term aim is to enable autonomous tool use in artificial systems (see 25,26 for recent publications). On the other hand, the work described in this paper can also be seen as embedding a robotic system with in situ primitive tool making abilities for a sensing purpose. To explore how the researches can benefit from each other is also part of our future work.

## Supporting Information

Video S1 **The implementation of autonomous in situ adjustment of sensor morphology and active sensing via suitable motion.** The video shows the process explained in Figure 4 and Figure 5 for discriminating objects softness and temperature based on the proposed approach.
(AVI)
